# Investigation of a Modular High-Pressure Heat Exchanger with Metal Foam Packing for a Pneumatic–Hydraulic Drive

**DOI:** 10.3390/ma17225557

**Published:** 2024-11-14

**Authors:** Roman Dyga, Sebastian Brol

**Affiliations:** 1 Department of Process Engineering and Environmental Engineering, Faculty of Mechanical Engineering, Opole University of Technology, Mikolajczyka 5, 45-271 Opole, Poland; 2 Department of Vehicles and Machines Mechatronics, Faculty of Mechanical Engineering, Opole University of Technology, Mikolajczyka 5, 45-271 Opole, Poland; s.brol@po.edu.pl

**Keywords:** open-cell metal foam, modular heat exchanger, pneumatic–hydraulic drive, heat exchanger efficiency

## Abstract

The results of the first stage of work aimed at improving a hybrid drive system in which the combustion engine is supported by a pneumatic–hydraulic motor are presented. The purpose of the described work was to show that a heat exchanger with a design adapted to the operating conditions of a pneumatic–hydraulic motor would allow sufficient air heating at the expense of waste heat from the combustion engine, thus increasing the efficiency of the drive system. It was assumed that the key component of the heat exchanger would be copper foam in order to increase the heat exchange surface. A prototype modular heat exchanger was designed and tested. An open-cell copper foam with a porosity of 0.9 and a pore density of 40PPI was placed in the heat exchanger. Experimental and numerical air heating studies were carried out under various heat exchanger operating conditions. The tests were conducted at initial air temperatures of −123 °C, −71 °C, and 22 °C and air pressures of 2.5 × 10^6^ and 7.0 × 10^6^ Pa. The air mass flux was in the range of 3.6–1644 kg/(m^2^s). It was found that the tested heat exchanger allows a reduction in air consumption in the drive system of 11% to 58% and increases the efficiency of the air expansion system by 16% to 30%. The maximum efficiency of the heat exchanger is 96%. The results of the work carried out will help to improve the pneumatic–hydraulic drive systems of work machines and vehicles.

## 1. Introduction

Machines and vehicles in many cases need a hydraulic drive system, which is designed to perform frequent or continuous operations. The hydraulic drive is also considered to be assistive or supportive to the main drive. This approach is observed in present-day hybrid vehicles, transport vehicles and machines, and machinery utilized in construction, as well as sea vessels and heavy transport systems. Regarding the driving aspect, the hydraulic system is considered as auxiliary, supportive, or fixed in parallel/series with respect to the main drive system.

This basic classification was recently improved by a new solution—the pneumatic–hydraulic drive system (PH system). It was initially introduced by Shaw et al. [1] and then improved by Brol et al. [2,3]. The PH system presented by Shaw and Brol, in all its variations, has potential applications in stand-alone, auxiliary, supportive, and hybrid drive and many varieties of its modes. The hybrid solutions of PH systems with combustion engines (ICE; i.e., fueled by alternative and hydrogen-like fuels) and electric engines are exploited in this study, due to their technical and technological novelty.

**Figure 1:**
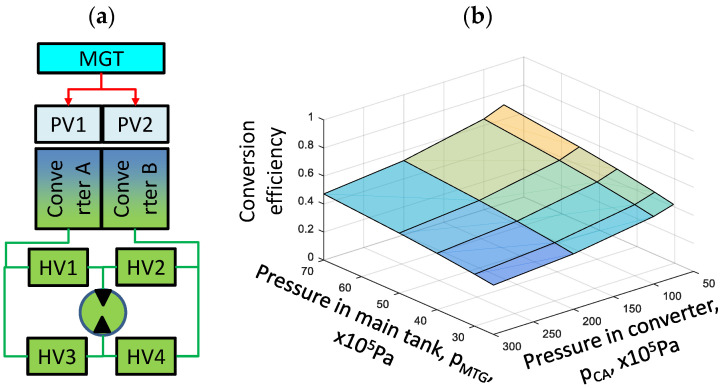
Pneumatic–hydraulic system (**a**) configuration; (**b**) conversion efficiency. MGT—main gas tank; PV—pneumatic valve; HV—hydraulic valve.

One such system was designed by Brol et al. [2], which includes the hydraulic motor shown in Figure 1a. The operation of such a drive unit is based on the alternating flow of hydraulic fluid between converters A and B. The pressure difference required to pump the fluid (shown in green in Figure 1a) is provided by compressed gas. The hydraulic valves HV1–HV4 ensure that the fluid flows in the chosen direction. The pneumatic–hydraulic system can operate in two modes: hybrid mode, when it cooperates with another engine, or as a stand-alone drive unit. In the latter case, the energy of the gas compressed in the main gas tank (MGT) is used. In both modes, there is rapid, almost adiabatic, gas expansion, which reduces its energy after expansion compared to when it was in its compressed state [2,4]. At the same time, the temperature of the gas decreases significantly—the temperature drop can exceed 100 K. The gas expansion efficiency of such a system is low and depends strongly on the pressures in the MGT and in the converters (Figure 1b).

Pressures are in the ranges of η_RG_ = 0.23 to η_RG_ = 0.83 for the MGT and (7.0–30.0) × 10^6^ Pa and (2.5–7.0) × 10^6^ Pa in the converters. This is the Achilles heel of the pneumatic part of PH systems. The shortest way to increase efficiency is to heat the decompressing gas. The best option here is to use waste heat.

A review of the literature indicates that there are a number of potential solutions to improve the efficiency of driving vehicles, which use a pneumatic hydraulic motor and a combustion or electric engine. There have been attempts to use a heat source to increase the gas temperature before the gas enters the pneumatic system. The most interesting sources of heat used were those generated from fuel burning [5], atmospheric heat [6], and even hot water [7]. However, to date, no one has tried to use oil from an ICE or oil from an electric propulsion system for this purpose. Moreover, in hybrid hydraulic vehicles with gas accumulators (a variant of an HP system with reduced capabilities, e.g., in terms of range), the waste heat exchange is omitted [8,9].

So, the idea investigated here is how the waste heat from an ICE or electrical drive could be used to improve the efficiency of a hybrid drive with an incorporated PH system. This is especially important considering the large amounts of waste heat generated in the process of vehicle acceleration, when the demand for energy is highest and, at the same time, a large part of it is affected by the non-ideal efficiency of coolant, air, or oil [4]. The best solution is to draw heat from the coolant, but the most available heated fluid (in ICE and electrical drives) is the engine oil.

Heating the air supply to the pneumatic motor using the oil heat from the main engine requires the use of a heat exchanger, which is designed to operate under extremely difficult conditions; namely, pressures up to 25 MPa and temperatures below −120 °C. Moreover, the heat exchanger must be light and small to maintain the aerodynamic properties of a vehicle [4,10].

The only known heat exchangers that meet these conditions are adhesion-welded plate exchangers. Thick metal plates with hollow channels for fluid flow give these exchangers great strength. This design, however, results in a relatively small heat transfer surface area and, thus, a limited heat transfer capacity. Increasing the heat transfer surface area of plate heat exchangers appears to be one of the most important factors for increasing the efficiency of a pneumatic motor hybrid drive. In the research described here, it was considered that open-cell metal foams could play a key role. These materials are characterized by a relatively large specific surface area with high porosity, usually exceeding 90%. Despite this high porosity, the value of the effective thermal conductivity coefficient of foams is relatively high due to the good thermal conductivity of the foam skeleton. These characteristics mean that metal foams are increasingly being used as elements that intensify heat transport in process apparatus. Foam is also used as a boiling promoter in the evaporators of refrigeration equipment, heat pumps, and solar collectors. Metal foams can be used in the construction of heat exchangers as a packing material to increase the heat transfer surface area.

The authors of studies on pneumatic–hydraulic drive units in the existing literature have, so far, not undertaken work to determine the impact of gas heating on the efficiency of these systems, nor have they described practical technical solutions for effectively heating gas in these systems. To the best of the authors’ knowledge, the improvement of the hybrid drive system described in this paper (consisting of an internal combustion engine supported by a pneumatic–hydraulic motor) by heating the air supplying the pneumatic motor at the expense of the waste heat of the engine oil has not been studied before. It is also a novelty, both in terms of the concept for operating the drive system and the design of the high-pressure adhesion-welded plate heat exchanger, which, for the first time, uses open-cell metal foam as a packing for the air channels.

Considering the above, our main interests in this study are as follows:

(1) How a heat exchanger with metal foams will influence the heat exchange characteristics when a vehicle is moving in a steady state (constant speed).
(2) How much media flow rates will affect the heat exchange in relation to the required fluid flow rates in vehicle transient states (acceleration).
(3) How the addition of a heat exchanger increases the PH drive efficiency of an innovative design.

Investigating all three topics is important to gain new knowledge, as the results of such an investigation are essential to develop this new pneumatic–hydraulic drive.

## 2. Drivetrain Concept

**Figure 2:**
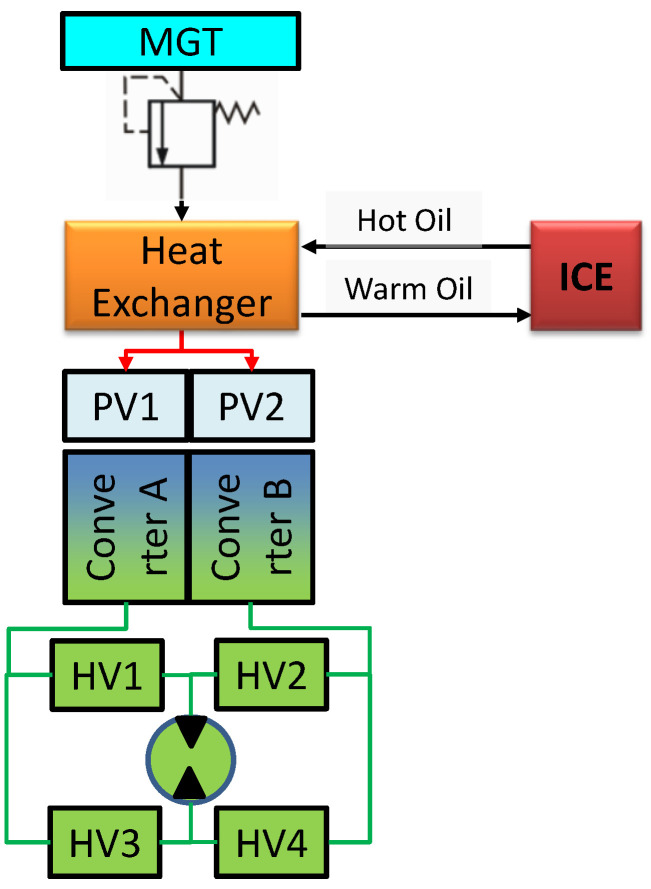
Pneumatic–hydraulic unit with a heat exchanger. ICE—internal combustion engine; MGT—main gas tank; PV—pneumatic valve; HV—hydraulic valve.

It was assumed that improving the efficiency of the hybrid drive unit could be achieved by heating the air expanding in the drive system of the pneumatic–hydraulic motor using the heat of hot oil in the lubricating system of the internal combustion engine. In a classic engine, this heat is partially lost. A schematic diagram of the drivetrain is shown in Figure 2. 

Air stored in the main gas tank (MGT) at a pressure of 25 × 10^6^ Pa due to the expansion of the pressure in converters A and B (2.5 × 10^6^–7.0 × 10^6^ Pa) can cool down to a temperature of 150 K. A heat exchanger located past the pressure regulator reduces this effect, which not only increases the efficiency of expansion but also increases the volume of the expanded air directed to the converters, thereby reducing the consumption of gas drawn from the MGT to drive the pneumatic–hydraulic motor. 

### Heat Exchanger

The heat exchanger must be designed to withstand high air pressures and with a small size, in order to facilitate its installation in vehicle drives. The amount of air needed to drive a pneumatic–hydraulic motor varies depending on the operating conditions of the drive, including the speed of the motor and the power required. For this reason, the design of the heat exchanger should allow its nominal thermal power to be varied. This ensures effective operation of the exchanger over a wide range of air temperatures for different values of air flow and pressure.

One of the basic assumptions was that an open-cell metal foam was needed to construct the exchanger. Numerous applications of metal foams have been cited by the authors of review articles [11,12,13,14,15]. Authors of works related to the use of metal foams in the construction of heat exchangers have compared traditional heat exchangers (usually shell-and-tube or plate heat exchangers) or finned surfaces, with the same designs, in which added metal foam fills the space that the fluid flows through or acts as specific fins.

Tamkhade et al. [16], as a result of numerical analysis of a double-tube heat exchanger (DTHE) with metal foams with pore densities ranging from 10 PPI to 50 PPI in which heat was transferred between two countercurrent water flows, found that as the pore density increased, both the heat transfer coefficient and pressure drop increased proportionally. In addition, they proved that placing the foam in an annular cross-section (between the inner and outer pipe) affects heat transfer more than when the foam is placed in a pipe with a circular cross-section. The authors of the paper [16] obtained good agreement between numerical results and solutions obtained based on empirical correlations taken from the literature.

Jadhav et al. [17] numerically analyzed heat transfer and flow resistance using a pipe partially filled with aluminum and copper foams. The fill occupied 40, 60, and 80% of the pipe’s diameter and consisted of two foams with pore densities of 20 and 45 PPI in three different configurations. The authors of this work found that using an infill that had foam with a higher pore density near the wall (heat transfer surface) than that in the center of the channel allows the heat transfer coefficient to be increased up to 23% compared to a that with uniform fill. At the same time, pumping power was found to decrease by up to 80%.

Hassan et al. [12] verified the performance of a crossflow heat exchanger in which foam was placed in the space between tubes. The authors of this work emphasize the importance of properly determining the foam’s parameters, including its thermal conductivity, permeability, porosity, and characteristic dimensions, due to their significant impact on both heat transport and pressure drop. In addition, they point out that the improvement in heat transfer depends on the thickness of the foam layer, and its correct determination requires optimization. Hassan et al. [12] point out that despite the undoubted improvement in the performance of metal foam heat exchangers, their high price may make such design solutions economically uncompetitive with other ways of increasing heat exchanger performance.

Dongellini et al. [18] conducted experimental tests on the thermal and hydraulic performance of three prototype water-to-air heat exchangers designed to cool electrical equipment. The prototypes were built using aluminum foam, which replaced the fins in traditional heat exchangers. The foam was attached to the tubes in various ways. The authors of the paper [18] found that the foam reduces air flow through the heat exchanger by up to 13% if it is connected to the tubes via thermally conductive paste and 42% if it is glued with epoxy glue. The heat transfer rate is also reduced by 60%, which, according to the authors of the paper in question, is due to the high porosity of the foam and the contact thermal resistance between the foam ligaments and the tubes. To reduce this resistance, Dongellini et al. [18] recommend soldering foam to the heat transfer surface.

Fiedler et al. present completely opposite conclusions to these studies [19]. They compared a heat exchanger in which an inner copper tube was surrounded by foam connected to the pipe by a single-step casting process. Water was pumped inside the pipe and through the foam. The authors [19] found that compared to an analogous shell-tube heat exchanger, heat transfer increased by 71% and heat flux density reached 30 kW. According to Fiedler et al., this value could be even higher, but in the exchanger studied, the heat penetration inside the tube, where no metal foam was placed, was insufficiently efficient. Kim et al. [20], when studying a plate heat exchanger in which they placed four types of metal foams (copper and nickel) between the plates, showed that when heating R245fa refrigerant, the Coulburn j factor could be as much as 6.3 times higher in the exchanger with foam than in the plate exchanger without foam.

Despite the ambiguous effect of foam on the efficiency and performance of heat exchangers, the prevailing opinion in the literature is that metal foams increase heat transfer. Such a view is presented, among others, by the authors of works [21,22,23,24,25]. Nevertheless, it should be emphasized at the same time that when considering the performance of heat exchangers with metal foams, the high drop in pressure cannot be ignored, as this reduces the energy efficiency of heat exchangers.

In the case of the heat exchanger designed as part of the work described in this article, this pressure drop is less important. Ultimately, the heat exchanger will be integrated into the air expansion system by throttling. The driving force for the air flow will be the pressure difference between the MGT and the hydraulic motor converters. Pushing air through the heat exchanger will therefore not require additional energy, e.g., for the compressor drive.

**Figure 3:**
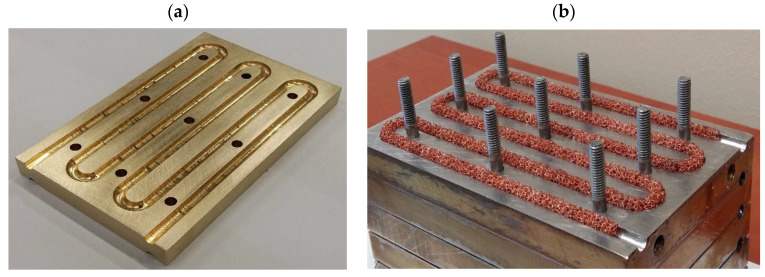
Heat exchanger design: (**a**) single plate; (**b**) stack of plates with foam-filled channel.

Taking into account the requirements specified at the beginning of Heat Exchanger, it was decided that the heat exchanger would take the form of a brass block in which winding channels placed on several horizontal planes were made. The channels are designed for the flow of air and oil. The compact design with a relatively thick wall should ensure the high strength of the exchanger and store a significant amount of heat, which can be important in the exchanger’s intermittent operation mode (typical of a hybrid drivetrain). The use of brass ensures the lowest possible resistance to heat conduction through the wall between the air and oil channels. The exchanger block is composed of symmetrical, repetitive flat plates (Figure 3a). On the lower and upper surfaces of each plate are milled channels with semicircular cross-sections (when the plates were assembled, channels of circular cross-sections were formed). The pair of adjacent air and oil channels and the plates forming them constitute a single module with a specific thermal power. By changing the number of plates, you can obtain a heat exchanger with any number of such modules. If the total heat output of the exchanger is not required, its reduction is relatively easy to achieve by cutting off the flow of fluids to individual modules. In the presented study, a heat exchanger with three modules was used. It was decided that metal foam infills would be placed only in the air channels (Figure 3b). This decision was based on the results of the works [26,27], where it was shown that in the case of fluid flow, the use of foams does not contribute to increased heat transfer.

The exchanger contains copper foam with a pore density of 40 PPI. The copper skeleton ensures the high thermal conductivity of the foam. The foam was soldered to the plates, which minimizes the thermal resistance between the foam skeleton and the channel surface. The specifications of the exchanger and the parameters and structure of the foam are shown in Table 1.

## 3. Test Stand, Scope, and Methodology of the Research

**Figure 4:**
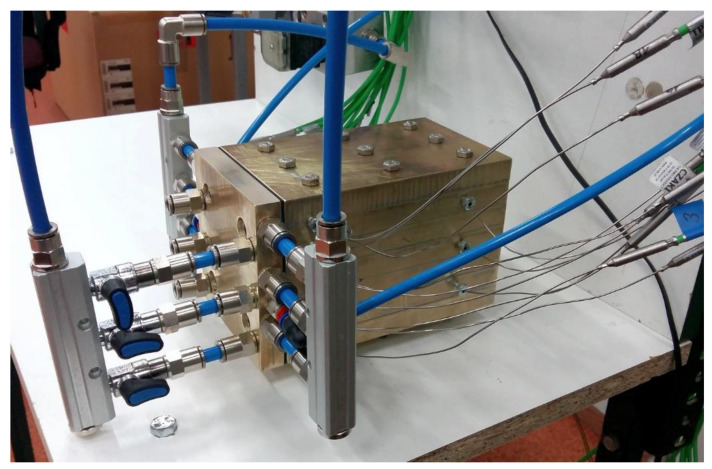
Prototype heat exchanger.

**Figure 5:**
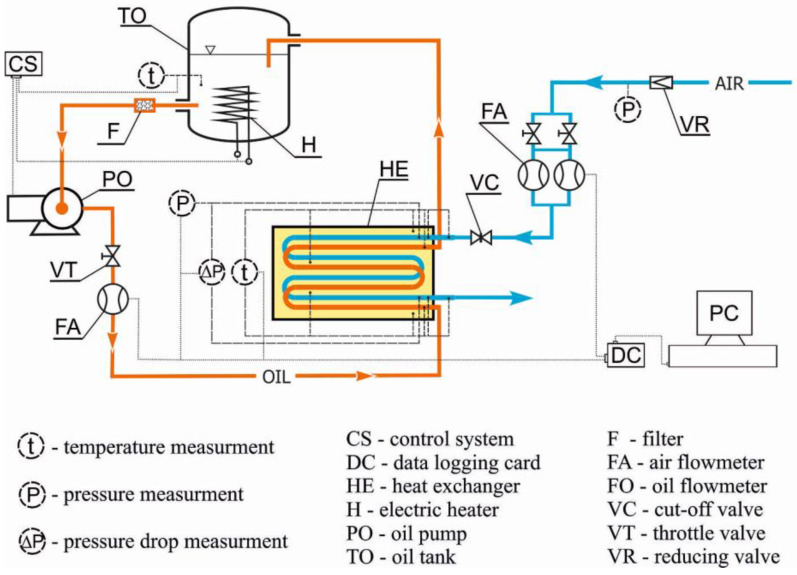
Schematic of the test stand.

The designed and built heat exchanger was subjected to tests in order to verify whether the developed design could be used to improve the hybrid drive unit. As part of the tests, the efficiency of air heating was determined under various operating conditions. At the current (preliminary) stage of this work, the heat exchanger was tested outside the hybrid drive unit using a laboratory test stand built for this purpose. A prototype of the exchanger is shown in Figure 4. A schematic of the test stand’s operation is given in Figure 5.

Air was taken from the pneumatic system. Velol-9Q oil (properties are given in Table 2) was fed to the exchanger from a 0.01 m^3^ tank, where it was heated using a 3000 W electric heater. After flowing through the exchanger, the oil was discharged back into the tank. Oil circulation was forced by means of a pump. Fluid fluxes were controlled with throttling valves, and their value was measured with flow meters. Two mass flow meters were used to measure air flow. Oil flow was measured using an oval gearwheel flow meter. As we needed to determine the air density, its overpressure at the heat exchanger inlet and the pressure drop of air flow through the exchanger were also measured. Piezoresistive pressure sensors were used for this purpose. The pressure drop was recorded as the pressure difference in the air channel immediately before and after the foam. The pressure drop in short, several-millimeter sections of the air channel at the inlet and outlet was negligibly small, compared to that in the foam.

**Figure 6:**
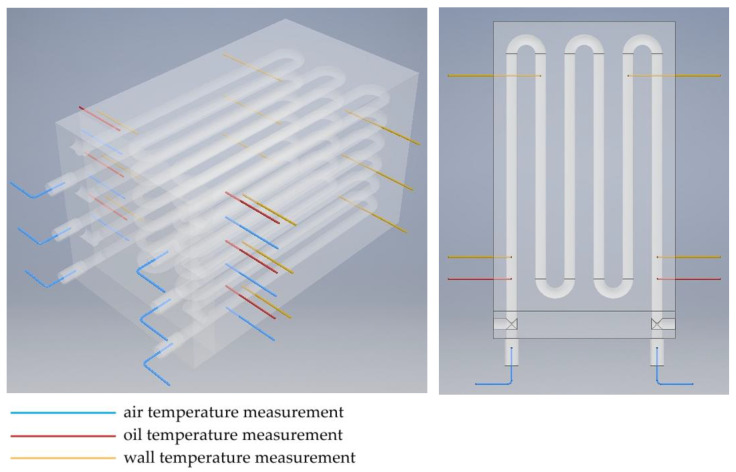
Location of thermocouples in the heat exchanger.

The oil temperature and the exchanger wall temperature were measured using K-type thermocouples with a diameter of 1 mm. Thermocouples with a diameter of 0.25 mm with a connector fused to the shell were used to measure air temperature. The low thermal inertia of these thermocouples allowed transient state testing. The measuring probes of the thermocouples measuring fluid temperatures were located in the axis of the individual channels, at the inlet and outlet of the heat exchanger. The temperature of the exchanger block was measured at 12 points lying on 3 planes located between the air and oil channels of each exchanger module. The location of all 24 temperature measurement points is shown in Figure 6. Thermocouples were connected by eight-channel measuring modules equipped with a reference temperature stabilization and correction system. Prior to testing, the temperature measurement system was calibrated. All thermocouples were checked at two fixed thermometric points (i.e., at the melting point of ice and the boiling point of distilled water). After calibration, the readings of all thermocouples were within ±0.2 K with respect to the reference temperature.

To maintain a constant oil flow and the desired oil temperature, the test stand was equipped with two independent open-loop control systems and a closed-loop control system. In programming the controller, programming methods, which were applied and tested in the measurement and control devices described in [29], among others, were used.

The signals of the measuring apparatus were monitored and recorded using a multi-channel measurement system developed in DasyLab. The measurement apparatus used in the testing had a correspondingly high accuracy. The specifications of the measuring apparatus are listed in Table 3.

The experimental results were evaluated in terms of measurement uncertainty *u*(*x*). The uncertainty analysis was carried out in accordance with the International Organization for Standardization (ISO) guidelines contained in the Guide to the Expression of Uncertainty in Measurement [30]. The standard uncertainty of quantity *x*, which was directly measured, is described by Equation (1):

(1) $$u\left(x\right)=\sqrt{\frac{{∆}_{d}x^{2}+{∆}_{e}x^{2}}{3}},$$

The value of calibration uncertainty ${∆}_{d}x$ was determined based on linear regression of the calibration results. The experimenter’s uncertainty ${∆}_{e}x$ was taken as the unit value of the last significant digit displayed in the result-recording system. Table 3 shows the maximum values of relative uncertainty (standard uncertainty versus the measured value) of particular measurement sensors.

## 4. Analysis of Experimental Results

The tests were conducted under steady-state and transient conditions. During the transient state tests, oil at a constant temperature was continuously pumped through the exchanger. Periodically, cold air was supplied to the exchanger for a period of several to several dozen seconds. Such conditions correspond to those occurring when an exchanger cooperates with a hydraulic engine in situations where the motor temporarily supports the combustion engine.

**Figure 7:**
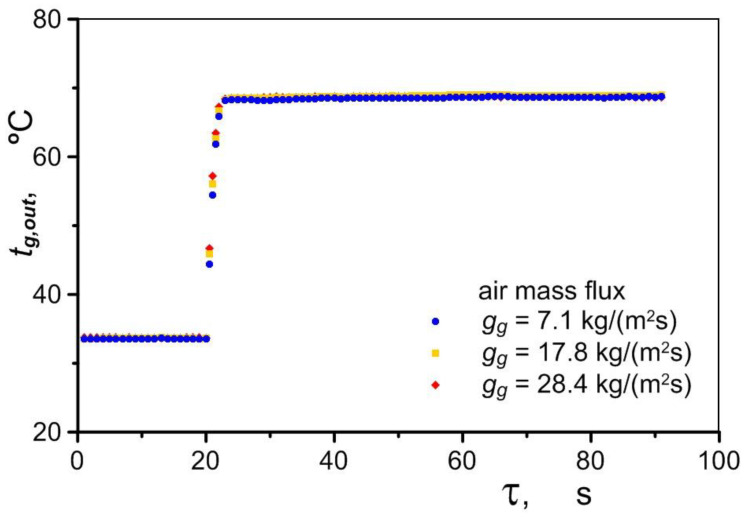
Change in air temperature at the outlet of the exchanger during periodic operation of the exchanger.

During the tests, a rapid increase in the final temperature of the air *t_g,out_* entering the exchanger was observed. The air reached its maximum temperature in less than 3 s in all cases tested (for different air flows), as shown in Figure 7. The temperature of the air flowing out of the exchanger was approximately 2 K lower than the temperature of the oil supplied to the exchanger, which indicates its high efficiency.

In tests carried out in the steady state, as in the transient state, a constant flow of oil and its temperature were maintained at the heat exchanger inlet. A constant flow of air was also supplied to the exchanger long enough for the system to reach equilibrium (i.e., when the air and oil temperatures at the exchanger outlet and the average block temperature were constant). Once the equilibrium state was reached and the test results were recorded, the value of the air flow was changed. Such conditions correspond to the cooperation of the exchanger with a hydraulic motor operating in a continuous manner. In addition, in the steady state, the quantities characterizing heat transfer in the exchanger can be determined more reliably (than in the transient state).

**Figure 8:**
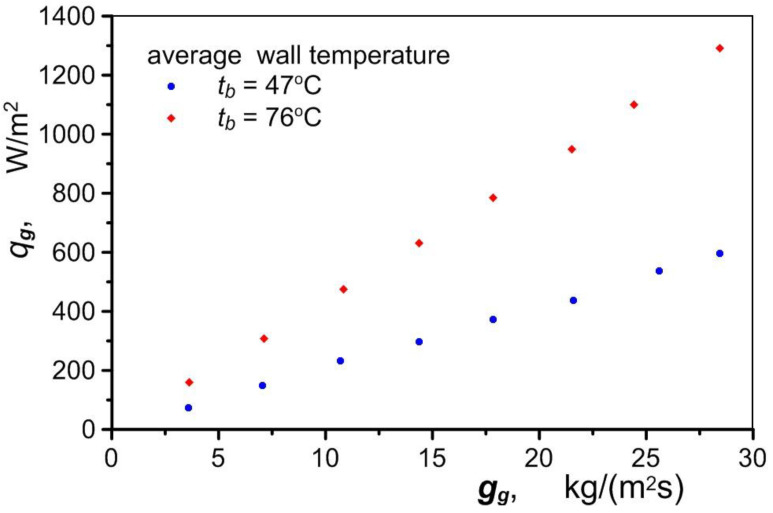
Effect of the heat exchanger temperature and air mass flow on the heat flux.

Figure 8 shows the heat flux density *q_g_* taken up by the air in the exchanger for two different average temperatures of the exchanger block ${\bar{t}}_{b}$. The relatively small value of the heat flux determined by Equation (2) is due to the heat that is transferred to a large heat transfer surface area. This surface area consists of the contact area of the air with the channel wall *A_ch_* and the area of the foam skeleton *A_sf_*,

(2) $$q_{g}=\frac{m_{g}c\left(t_{g,out}-t_{g,in}\right)}{A_{ch}+A_{sf}}.$$

The foam area is the product of the volume occupied by the foam and the foam’s specific surface area *a_sf_*, which was determined using Equation (3), as recommended by many authors of works on metal foams, such as [17,31,32,33]:

(3) $$a_{sf}=\frac{3\pi d_{l}}{{\left(0.59d_{p}\right)}^{2}}\left(1-e^{-\left(1-\varepsilon \right)/0.04}\right),$$

where the diameter of the skeleton ligament *d_l_* is described by Equation (4) developed by [34]:

(4) $$\frac{d_{l}}{d_{p}}=\frac{1.18}{1-e^{-\left(1-\varepsilon \right)/0.04}}\sqrt{\frac{1-\varepsilon }{3\pi }},$$

in conjunction with the pore diameter *d_p_*:

(5) $$d_{p}=\frac{0.0254}{PPI}.$$

**Figure 9:**
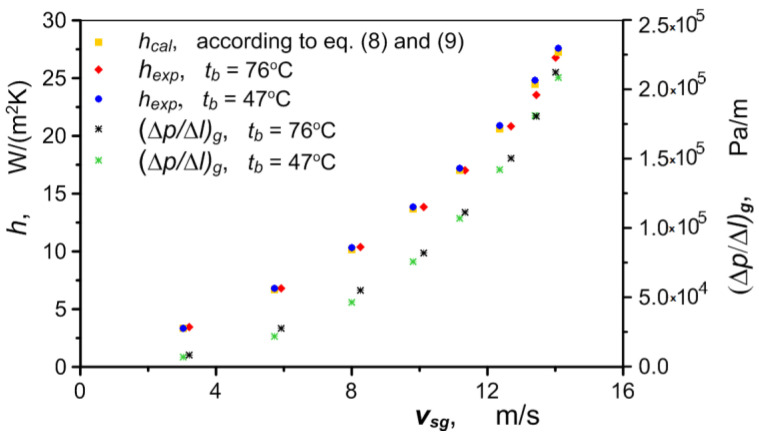
Heat transfer coefficient and pressure drop in air flow through a heat exchanger versus air velocity.

As can be seen in Figure 8, the heat flux taken up by the air clearly depends on the wall temperature ${\bar{t}}_{b}$, as well as the air flux. The wall temperature has no effect on the intensity of the heat transfer. Figure 9 shows a clear, almost exponential effect of the air velocity on the value of the heat transfer coefficient *h_exp_* (regardless of the temperature ${\bar{t}}_{b}$). The nature of the variation in this coefficient is due to the direct correlation between air velocity and pressure. The pressure drop ∆*p/*∆*l* through metal foams is relatively high. The pressure in the exchanger rises sharply as the air flow increases, compressing the air and causing a disproportionately small increase in velocity compared to the increase in the flow. The analogous nature of changes in the pressure drop and heat transfer coefficient was recorded during the tests (Figure 9).

The experimental value of the heat transfer coefficient *h* was determined based on the equation

(6) $$h=\frac{q_{g}}{{\bar{t}}_{b}-{\bar{t}}_{g}},$$

in which the average gas temperature is calculated based on the air temperature at the inlet *t_g,in_* and outlet *t_g,out_* of the exchanger:

(7) $${\bar{t}}_{g}=0.5\left(t_{g,in}+t_{g,out}\right).$$

The average temperature of the exchanger block, determined based on the readings of four thermocouples placed in a specific exchanger module, was used as the heat transfer surface temperature needed to calculate the heat transfer coefficient.

Statistical analysis of the collected measurement data made it possible to describe the heat transfer in the tested exchanger with the Nusselt equation in the form of

(8) $$Nu=0.74\times 10^{-3}{Re}_{dl}^{1.01}{Pr}^{0.37}.$$

where

(9) $$Nu=\frac{hd_{l}}{k_{g}},$$

(10) $${Re}_{dl}=\frac{v_{sg}d_{l}\rho_{g}}{\mu_{g}},$$

Referencing the Nusselt number and Reynolds number for the dimensions of the skeleton ligament is recommended by many researchers [16,35,36], especially when heat transfer cannot be treated as an equilibrium process (i.e., when the temperature of the fluid and the solid and their thermal conductivity differ significantly).

**Figure 10:**
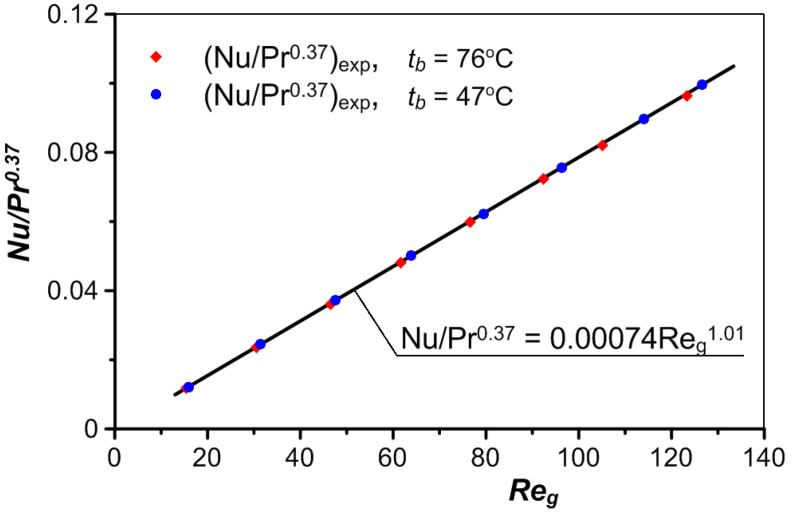
The Nusselt equation compared to the experimental data.

The values of the heat transfer coefficient *h_cal_* determined based on Equations (8) and (9) differ by no more than 2% from the values of *h_exp_*, obtained experimentally. A comparison of these values is presented in Figure 9. The values of the Nusselt number *Nu* calculated using Equation (8) and the corresponding values calculated based on *h_exp_* are shown in Figure 10.

Experimental results indicate that the efficiency of air heating was high (the maximum temperature of the heated medium was close to the maximum temperature of the heating medium), but the tests were carried out for an initial air temperature of approximately 22 °C and a pressure not exceeding 3 × 10^5^ Pa, which is very different from the operating conditions of the exchanger in the drivetrain. The temperature of 22 °C was chosen based on previous road tests carried out on a prototype vehicle with a pneumatic–hydraulic drive. This was the temperature in the converter just after starting the car and during two cycles. A similar temperature of 18–24 °C was recorded just after braking with gas compression in the converters.

To avoid damaging the exchanger, tests for low temperatures and high pressures were carried out by means of numerical simulations. The objective of the numerical work was to evaluate the efficiency and performance of the heat exchanger under conditions not tested experimentally.

## 5. Simulation Conditions and Results

**Figure 11:**
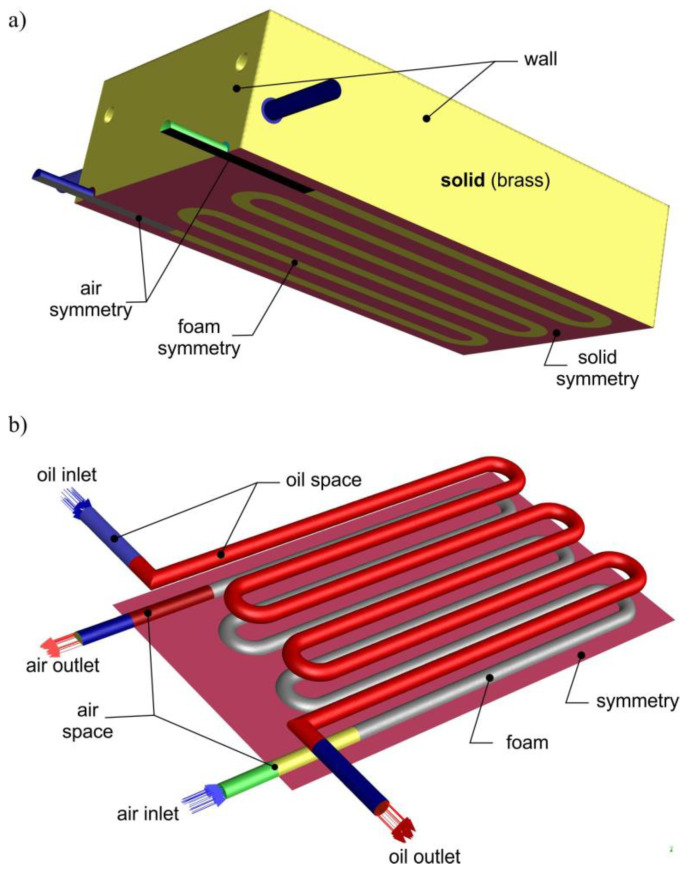
Numerical simulation domain: (**a**) external surfaces, (**b**) fluid space.

Numerical simulation of the heat exchanger’s operation was carried out using Ansys Fluent 2021 R2 software and the geometric model of the exchanger prepared for this purpose, which is shown in Figure 11. In the three-module heat exchanger studied, the center air channel lies on the exchanger’s horizontal plane of symmetry, which made it possible to limit the computational domain to half of the exchanger. The Neumann condition was applied for the plane of symmetry, according to which the velocity gradient in the direction normal to the plane of symmetry is zero: $\frac{\partial u_{x}}{\partial n}=\frac{\partial u_{y}}{\partial n}=\frac{\partial u_{z}}{\partial n}=0$. On the surface of the channels, the absence of fluid slip was assumed (fluid velocity components *u_x_ = u_y_ = u_z_* = 0). A heat convection of 12 W/(m^2^ K) was assumed for all external surfaces of the solid. A constant mass flow (*m* = *const.*) and constant fluid temperature (*t = const*.) were set at the inlets to the channels. The fluids flowed out into a space of known pressure. For oil, the ambient pressure was assumed, while for air, the pressure *p_AB_* prevailing in the converters of the hydraulic motor was used. Where the fluids contacted the walls of the exchanger, the continuity of temperature and heat flux density was valid. Since the foam ligaments were soldered to the exchanger block, the thermal resistance at the contact points of these materials was ignored.

A simulation of the flow of oil and air in the hollow region (outside the metal foam) was carried out based on the turbulence model *k-ω* SST, which allowed us to obtain an accurate solution in both the fluid boundary layer and the turbulent flow region. This necessitated the generation of a dense grid near the channel wall such that the parameter Y^+^ ≈ 1.

The equations governing the flow, according to the adopted model, are as follows:

The continuity equation:

(11) $$\frac{\partial \rho }{\partial t}+\nabla \left(\rho u_{i}\right)=0,\;i=x,\;y,\;z$$

The momentum equation:

(12) $$\frac{\partial u_{i}}{\partial t}+u_{j}\frac{\partial u_{i}}{\partial x_{i}}=-\frac{1}{\rho }\frac{\partial p}{\partial x_{i}}-\frac{1}{\rho }\frac{\partial }{\partial x_{j}}\left(\left(\mu +\mu_{t}\right)\left(\frac{\partial u_{i}}{\partial x_{j}}+\frac{\partial u_{j}}{\partial x_{i}}\right)\right),$$

The turbulent kinetic energy transport equation k and turbulent specific dissipation rate: *ω*

(13) $$\frac{\partial }{\partial t}\left(\rho k\right)+\frac{\partial }{\partial x_{i}}\left(\rho ku_{i}\right)=\frac{\partial }{\partial x_{j}}\left(\Gamma_{k}\frac{\partial k}{\partial x_{j}}\right)+G_{k}+Y_{k}+S_{k},$$

(14) $$\frac{\partial }{\partial t}\left(\rho \omega \right)+\frac{\partial }{\partial x_{j}}\left(\rho \omega u_{j}\right)=\frac{\partial }{\partial x_{j}}\left(\Gamma_{\omega }\frac{\partial \theta }{\partial x_{j}}\right)+G_{\omega }-Y_{\omega }+D_{\omega }+S_{\omega },$$

where *G_k_* represents the generation of turbulence kinetic energy, and *G_ω_* is the production of the specific dissipation rate. Other quantities and the equations describing them are explained in detail in [37].

The channel space that contained the metal foam was treated as a porous zone, with a homogeneous and isotropic structure. The Forchheimer–Brinkman-extended Darcy model, which takes into account viscosity and the inertial effect, was used to model the fluid flow in this space. According to this model, the momentum equation in the foam region is:

(15) $$\frac{\rho_{f}}{\varepsilon^{2}}\left(\vec{V}\cdot \nabla \right)\vec{V}=-\nabla {\langle P\rangle }_{f}+\mu_{f,eff}\nabla^{2}\vec{V}-\left(\frac{\mu_{f}}{K}+\frac{\rho_{f}C_{f}}{\sqrt{K}}\left|\vec{V}\right|\right)\vec{V},$$

In this equation, permeability *K* and the inertial coefficient *C_f_* are quantities specific to a given metal foam. Their values were determined based on flow resistance studies.

Heat transfer in the flow of air through metal foam was treated as a process described by the local thermal non-equilibrium model, according to which the temperatures of the fluid and the solid are different. The energy equation for a fluid takes the form

(16) $${\left(\rho c\right)}_{f}\left(\vec{V}\cdot \nabla T\right)=\nabla \left(\varepsilon k_{f}\nabla T\right)+h_{sf}a_{sf}\left(T_{s}-T_{f}\right),$$

and for a solid,

(17) $$0=\left(1-\varepsilon \right)\nabla \cdot \left(k_{s}\nabla T_{s}\right)-h_{sf}a_{sf}\left(T_{s}-T_{f}\right),$$

According to many researchers (e.g., [31,38,39,40,41]), in the case of flow through foams, the local thermal non-equilibrium (LTNE) model describes heat transfer much better than the thermal equilibrium (LTE) model, which is usually used for other porous zones. According to [42], for flow through copper foam, the heat transfer intensity calculated based on the LTE model can be more than 20% higher than that calculated using the LTNE model.

The interfacial convection heat transfer coefficient *h_sf_* (in Equations (16) and (17)) is described according to correlations (18) proposed by Zhukauskas [43] and considered in [37].

(18) $$h_{sf}=\left\{\begin{array}{c} 0.76{Re}_{dl}^{0.4}{Pr}^{0.37}\frac{k_{f}}{d};\;\;\left(1\leq {Re}_{dl}\leq 10\right)\;\\ 0.52{Re}_{dl}^{0.5}{Pr}^{0.37}\frac{k_{f}}{d};\;\;\left(40<{Re}_{dl}\leq 10^{3}\right)\\ 0.26{Re}_{dl}^{0.6}{Pr}^{0.37}\frac{k_{f}}{d};\;\left(10^{3}<{Re}_{dl}\leq {2\times 10}^{5}\right) \end{array}\right.,$$

The interfacial surface area *a_sf_* is described by Equation (3), consistent with [37].

Oil was treated as an incompressible fluid, while air was treated as a real gas described by the Soave–Redlich–Kwong model. The physical properties of liquids and solids were made dependent on temperature changes.

The initial and boundary conditions corresponded to the assumed operating conditions of the hybrid drivetrain, operating at varying speeds of the hydraulic motor in the range of 200–1800 rpm (the hydraulic motor is a slow-speed unit). Each engine speed corresponds to a well-defined flow rate of air.

Simulations were carried out for nine speeds *n* at the two most unfavorable variants of system operation. The first assumed the expansion of air from the initial pressure (in the MGT) of 250 × 10^5^ Pa to the pressure prevailing in the converters of the hydraulic motor *p_AB_* equal to 25 × 10^5^ Pa. According to the authors of the paper [1], this is the minimum pressure at which the hydraulic motor can properly operate. In this case, the air directed to the exchanger had the lowest temperature *t_g,in_* = −123 °C. In the second variant, the pressure in the converters *p_AB_* = 70 × 10^5^ Pa was assumed, which is optimal for the hydraulic motor [1]. At this pressure, the initial temperature of the air *t_g,in_* = −71 °C, so the air did not need to be heated as much as in the first variant, but for a certain engine speed, the mass of the heated air was then much greater due to its higher density. Under the conditions adopted for the numerical studies, the parameters characterizing the operation of the heat exchanger reached minimum and maximum values. Under other conditions, these parameters take intermediate values.

The post-expansion air temperature and pressure in the hydraulic engine converters were set as boundary conditions at the air inlet and outlet, respectively. The oil flow rate and the oil initial temperature in each case analyzed were the same; namely, temperature *t_ol,in_* = 95 °C and flow rate *m_ol_* = 13 × 10^−3^ kg/s. The conditions of the simulated cases are summarized in Table 4.

**Figure 12:**
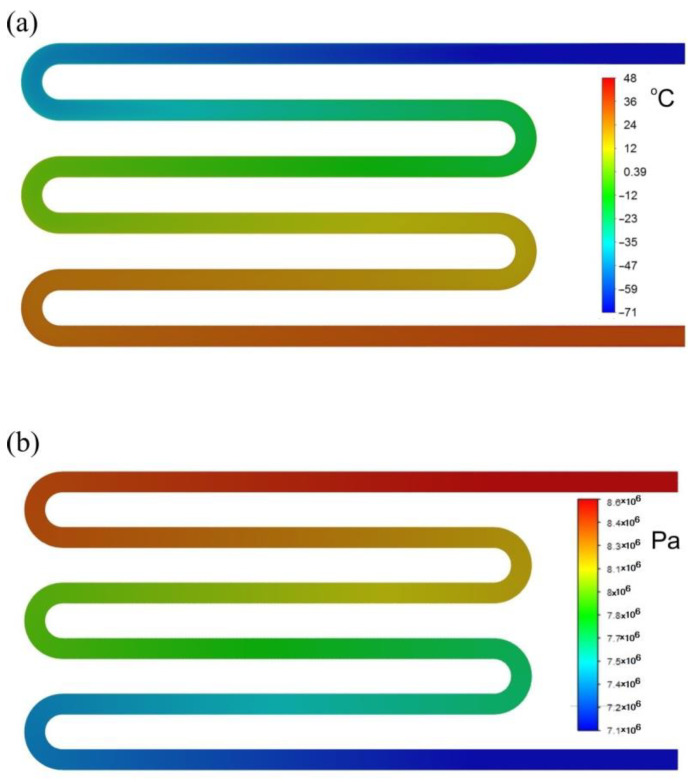
Simulation result for variant W-II.600: (**a**) temperature distribution; (**b**) pressure distribution.

Figure 12 shows the distributions of velocity and pressure in the air channel’s center plane, which were typical of all cases analyzed. A gradual increase in air temperature along the channel is clearly visible, with a simultaneous decrease in pressure.

It was assumed that the temperature of the air driving the hydraulic motor should be above 0 °C. Heating the air to a positive temperature prevents operational problems associated with frosting of pneumatic system components and, more importantly, does not lead to excessive cooling of the oil in the hydraulic engine converters. When the oil temperature is too low, it is difficult to pump the oil between the converters.

**Figure 13:**
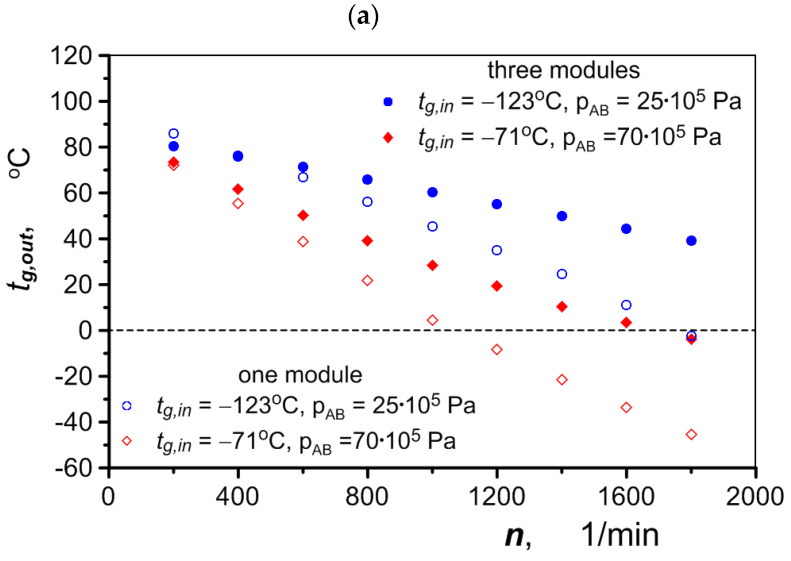

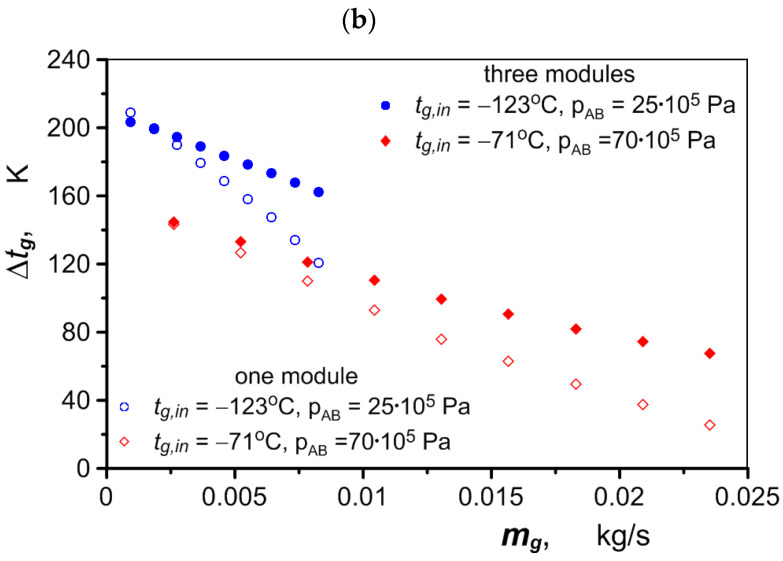
Effects of air heating when flowing through one versus three heat exchanger modules: (**a**) air temperature at exchanger outlet; (**b**) increase in air temperature.

The flow of air heated in the exchanger and its final temperature, *t_g,out_* (at the outlet of the exchanger), depend on the operating parameters of the hydraulic motor—mainly its speed and the pressure in the converters. When the pressure in the converters is at its lowest, 25 × 10^5^ Pait, it is possible to heat the air to a positive temperature over the entire range of engine rotational speed using only one exchanger module (air is pumped through one channel only). At the same rotational speeds and the highest possible pressure in the converters, *p_AB_* = 70 × 10^5^ Pa, the mass flow of air is much higher due to the gas density being almost three times higher. This is reflected by the much lower final air temperature. When only one exchanger module is used, the air reaches a positive temperature if the engine speed is no higher than 1000 rpm. At higher speeds, the air flow increases to such an extent that more exchanger modules must be used to heat the air. The temperature of the air after heating under different exchanger operating conditions is shown in Figure 13a, as a function of the speed of the hydraulic motor. The increase in air temperature as a function of air mass flow rate is shown in Figure 13b.

**Figure 14:**
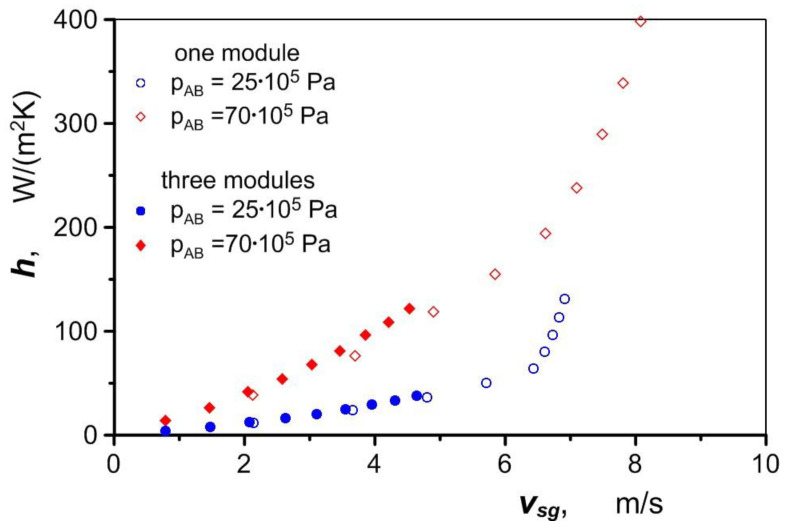
Heat transfer coefficient for heating air in one versus three heat exchanger modules.

As can be seen in these figures, the difference in the air heating using one versus three exchanger modules increases as the air flow rate increases. Air pumped through one module flows at approximately three times the velocity. The higher velocity in a single channel increases the intensity of heat transfer compared to the flow in three channels (Figure 14), which compensates for the smaller heat transfer area of a single module, and as a result, the final temperature of the air can be similar in both cases.

**Figure 15:**
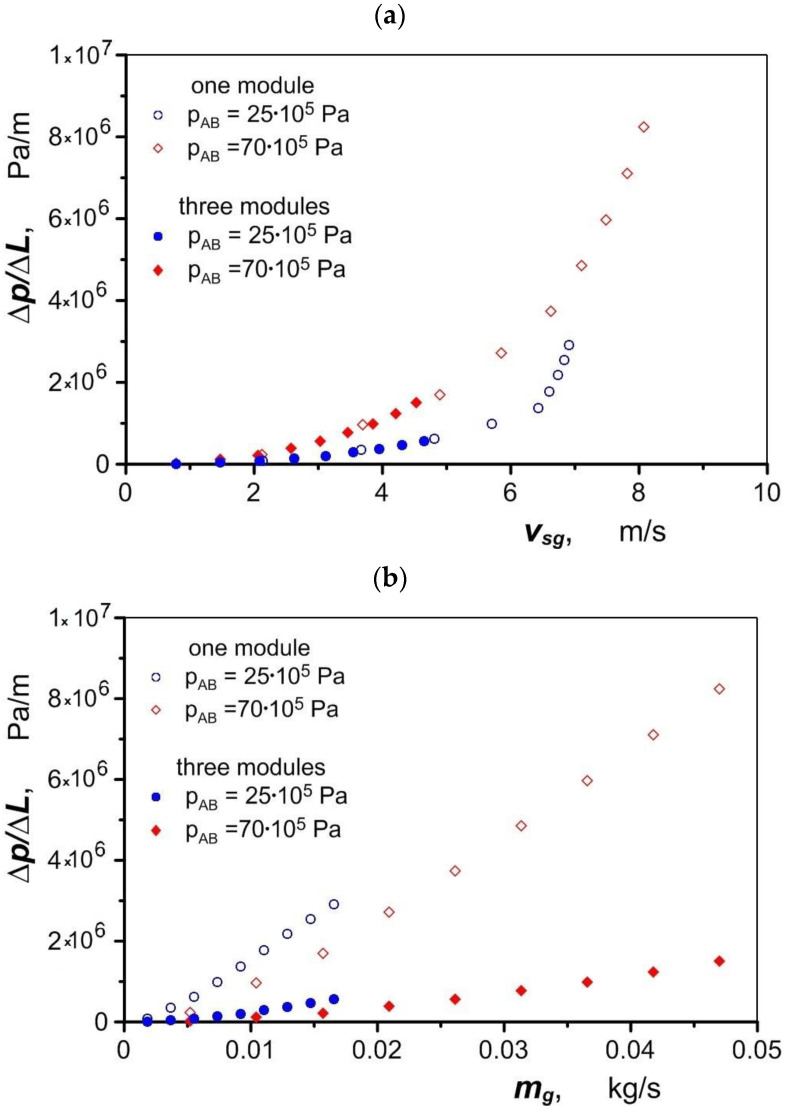
Air pressure drop in the heat exchanger: (**a**) as a function of air velocity; (**b**) as a function of mass flow rate.

As the air velocity increases, the pressure drop increases sharply (Figure 15a), as does the heat transfer coefficient. The compressibility of the gas causes its velocity and heat transfer coefficient to increase less than the mass flow. As the gas flow increases, this effect intensifies and the role of the heat transfer surface increases, so using more modules allows the air to be heated to a greater extent compared to when it flows through a single module.

Splitting the air flow into three modules results in a reduction in the air flow pressure drop compared to single-channel flow by as much as 67.5 × 10^5^ Pa (Figure 15b). To achieve stable operation of the drive, a constant pressure must be maintained in the pneumatic–hydraulic motor converters located downstream of the heat exchanger. The air pressure before the entry to the heat exchanger should therefore be set (in the expansion system) taking into account the pressure drop across the exchanger.

**Figure 16:**
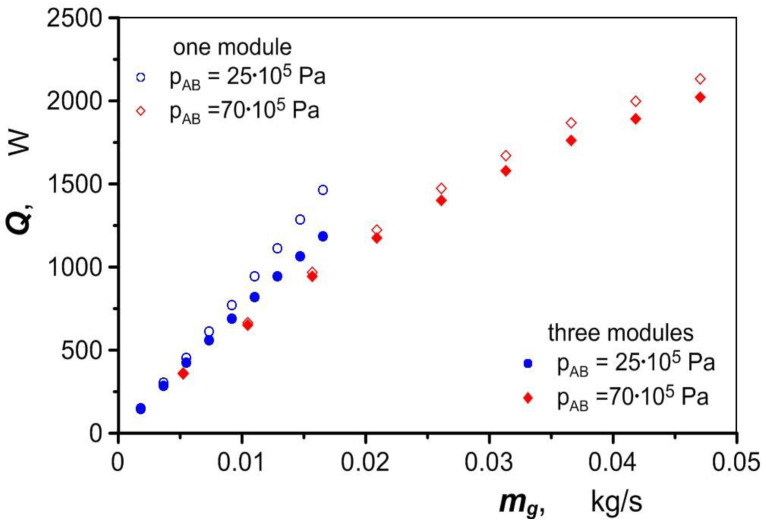
Variation in heat exchanger power as a function of air mass flow.

**Figure 17:**
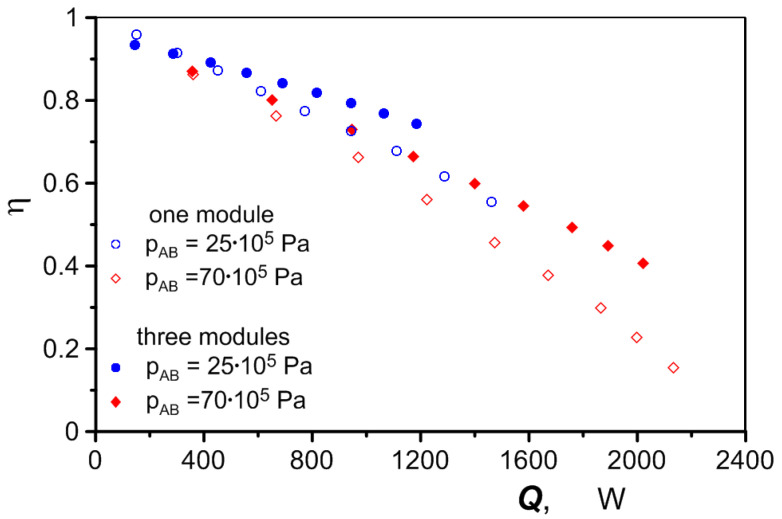
Heat exchanger efficiency in relation to its performance and number of modules.

When using one heat exchanger module, its performance is several percentage points higher than when three modules are used (Figure 16). The larger heat exchange surface area of a heat exchanger with three modules results in a higher air heating efficiency (an increase in its temperature), which is reflected in the efficiency of the heat exchanger. The efficiency of the heat exchanger *η*, as defined by the equation

(19) $$\eta =\frac{t_{g,out}-t_{g,in}}{t_{ol,in}-t_{g,in}},$$

exceeds 0.9 when the heat exchanger has a poor performance and decreases rapidly when it performs well (Figure 17). The reduction in the exchanger efficiency at high flow rates is due to the reciprocal relationship between the heat transfer intensity and pressure drop. As the air flow rate increases, the air velocity and pressure drop increase. This causes the air to expand to a lower pressure, which corresponds to a lower temperature. As a result, the final air temperature *t_g,out_* decreases. At a constant oil temperature *t_ol,in_*, the relationship between the numerator and denominator in Equation (16), which describes the efficiency of the heat exchanger, becomes less favorable as the air flow rate increases. The situation is improved by distributing the air flow over a greater number of heat exchanger modules, which reduces the adverse effect of the phenomenon described above. According to the results shown in Figure 17, the efficiency of the heat exchanger drops from 96% to 15% when the air is heated in one module and from 93% to 41% when three heat exchanger modules are used.

Air heated in the exchanger has an increased volume due to the temperature increase and pressure drop. A certain gas volume flow rate is required to drive the hydraulic motor, regardless of its pressure. An increase in the volume of air in the exchanger therefore results in a reduction in the amount of gas drawn from the high-pressure tank compared to the air consumption of a drivetrain without a heat exchanger. The reduction in gas consumption *δV_g_* is defined by the equation

(20) $${\delta V}_{g}=\left(1-\frac{\rho_{g,out}}{\rho_{g,AB}}\right)\cdot 100\%,$$

where *ρ_g,AB_* is the density of air at the pressure *p_AB_* existing in the converters of the hydraulic motor at the temperature resulting from the expansion of the air to a pressure of *p_AB_*.

**Figure 18:**
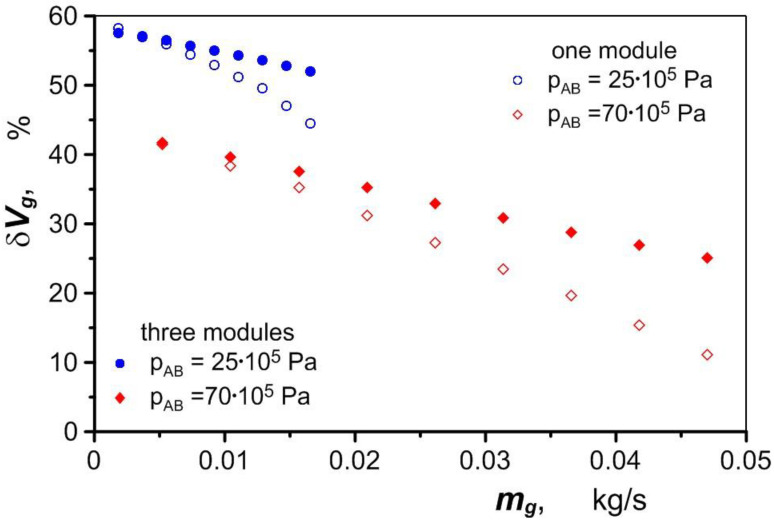
Reduction in drivetrain air consumption under various operating conditions.

As shown in Figure 18, the use of a heat exchanger in an pneumatic–hydraulic hybrid drive reduces the amount of air consumed by 11% to 58% depending on the operating conditions of the drivetrain. Greater benefits can be achieved if all three heat exchanger modules are used.

**Figure 19:**
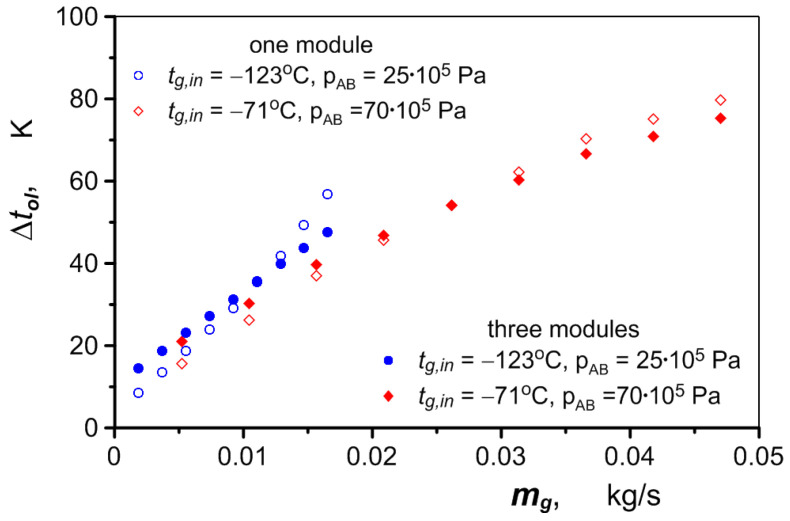
Temperature change of the heating oil in the heat exchanger.

Heating the air to the required temperature involves a significant drop in oil temperature, especially when the air flow is relatively large, as illustrated in Figure 19. In an extreme case, the oil temperature can drop by as much as 80 K to 14 °C. This is undesirable from the point of view of operating an internal combustion engine with a hydraulic motor. The oil temperature in the lubrication system should be maintained at a sufficiently high level to maintain the required lubricating properties. For this reason, heating of the air must not take place when the hydraulic motor is running continuously. When the hybrid drive is used in short cycles, such as during a momentary increase in the load of the internal combustion engine during acceleration, the heat necessary to heat the air is taken first from the body of the exchanger, which, due to its large mass, has a significant heat capacity. The oil temperature changes are smaller then. The air can be heated continuously when the hydraulic motor operates at low performance (low speed and operating pressure). The amount of oil directed to the heat exchanger can then be reduced, which keeps the oil temperature in the internal combustion engine at the required level and does not allow the temperature of the air supplying the hydraulic motor to rise unnecessarily above the ambient temperature. At the same time, this reduces the risk of overheating the hydraulic motor, because, as shown in Figure 13a, the air temperature can rise above 80 °C in the heat exchanger.

**Figure 20:**
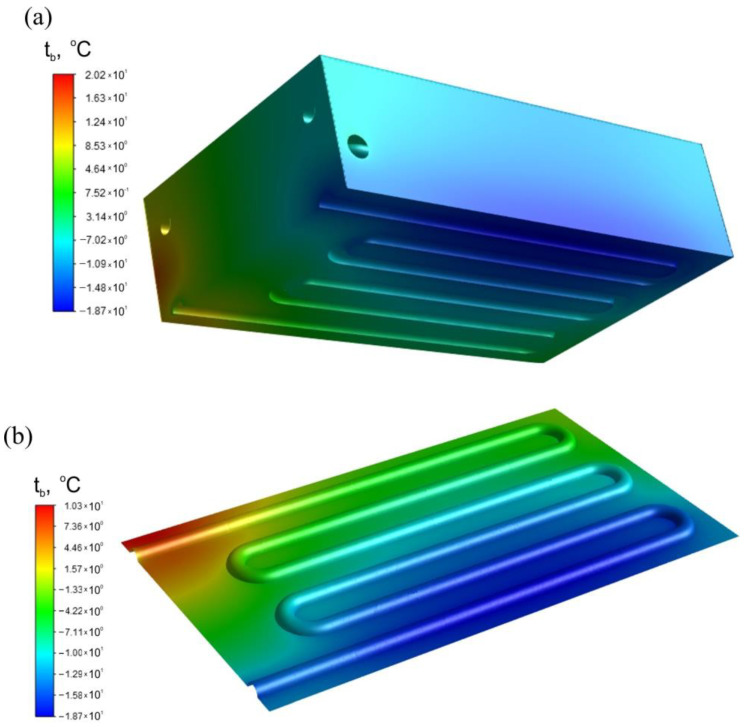
Temperature distributions: (**a**) heat exchanger block; (**b**) surface of the air channel.

Figure 20 shows the temperature distribution of the heat exchanger block for the W-I.2000 variant, where the average block temperature was the lowest due to the low initial air temperature (−123 °C) and relatively high gas mass flow. The block material takes a minimum temperature at the air inlet, where the surface of the air channel has a temperature of −18.7 °C. At this temperature, the brass does not manifest the characteristics of a brittle material, so it can be assumed that the exchanger will be safe to use.

## 6. Conclusions

The described research carried out under laboratory conditions allowed a preliminary evaluation of the adopted concept aimed at increasing the efficiency of a hybrid drivetrain before conducting road tests of the drive. The data necessary to analyze the strength of a heat exchanger exposed to ultra-low temperatures and high-pressure gas were also obtained.

The basis of the work carried out was the assumption that the energy losses associated with the expansion of air in the supply system of the pneumatic–hydraulic motor can be offset by heating the air with the oil from the internal combustion engine.

The heat exchanger required for this purpose was designed as a set of plates shaped and positioned in such a way that alternating channels for air and oil flow were formed. Metal foam was placed in the air channels to increase the heat transfer surface area. Channels in two adjacent plates formed an individual exchanger module. Metal foam placed in the heat exchanger channels increased the heat exchange surface 8.8 times compared to that for a heat exchanger without foam. The modular design of the heat exchanger allowed its surface and power to be adjusted to the changing operating conditions of the drive system.

Despite the low initial air temperature, the exchanger plates do not cool below −19 °C. Thus, no problems associated with low-temperature embrittlement of the material should occur during operation of the exchanger. The relatively thick plates should provide the required resistance of the exchanger to high pressure. The pressure distribution and temperature field of the heat exchanger block, obtained numerically, will be the basis for analysis of the strength of the heat exchanger before further testing of the exchanger in a real drivetrain is undertaken.

The results of the tests carried out allowed us to conclude that the design of the heat exchanger enables sufficiently effective heating of the air. The air is heated during expansion in the pneumatic part of the PE system from 20 to 210 degrees, depending on the operating conditions of the pneumatic–hydraulic motor, which determine the initial temperature of the air, its pressure, and mass flow.

Regarding the three topics considered, the investigation revealed the following facts:

(1) Regarding the heat exchanger’s characteristics, it was found that its efficiency is not constant but diminishes when mass flow through the exchanger increases. This regularity does not apply to the exchanger’s overall performance, as the output temperature increases with flux but at the cost of reduced efficiency. The efficiency of the heat exchanger drops from 96% (for the smallest air flow—1.8 × 10^−3^ kg/s) to 15% (for the largest amount of air—47 × 10^−3^ kg/s) when the air is heated in one module and from 93% to 41% when three heat exchanger modules are used. To achieve the highest possible efficiency of the drivetrain, the method of air distribution to individual exchanger modules should be correlated with the control of the pneumatic–hydraulic motor and the air expansion pressure controller. Control procedures for the air expansion system should take into account the large air pressure drop in the exchanger to ensure proper pressure levels in pneumatic–hydraulic converters.
(2) During acceleration of the vehicle, when the heat exchanger operates periodically (with momentary operation of the pneumatic–hydraulic system in the drivetrain), the time to reach the maximum air temperature is less than 3 s. The warm-up time is shorter than the time needed to fill up the converter with gas at the highest vehicle velocity (which is 6.3 s). Here, ‘the highest vehicle velocity’ means the highest fluxes in pneumatic and hydraulic subsystems.
(3) As air flows through the exchanger, it expands due to the pressure drop and temperature rise. As a result, the air consumption of the pneumatic–hydraulic motor can be reduced by 58% compared to the unheated air flow. Based on earlier analysis of the efficiency of the gas expansion system (Brol et al. [1]), it can be concluded that heating the expanded air to a temperature equal to the air temperature in the high-pressure tank (MGT) increases the efficiency of the expansion system by at least 16–30%, depending on the operating conditions of the pneumatic–hydraulic motor in the drive system. Heating the air results in a lower oil temperature in the internal combustion engine; for this reason, the pneumatic–hydraulic engine should be used in short cycles during increased demand for propulsion power.

## Figures and Tables

**Table 1:** Specifications of the exchanger with the infill.

**Exchanger parameters**		
external dimensions, *L* × *S* × *H* [m].	0.175 × 0.100 × 0.105	
number of plates	7	
number of air–oil modules	3	
diameter of channels, *d* [m]	0.006	
length of a single channel, *l* [m]	0.905	
heat transfer area of a single module, *A_ch_* * [m^2^]	1.706 × 10^−2^	
**Foam parameters**		
pore density, *ω* [PPI].	40	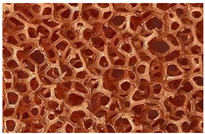
porosity, *ε*	0.9	
effective thermal conductivity, *k_eff_* [W/(m·K)]	38.9	
permeability, *K* ** [m^2^]	1.464 × 10^−7^	
inertial coefficient, *β* ** [m]	534.3	

* The surface of the air channel excluding the surface of the foam skeleton. ** quantities determined in accordance with the methodology described in [28], based on measurements of pressure drop in a straight channel 0.2 m in length.

**Table 2:** Characteristics of Velol-9Q oil.

Property	Dependence on Temperature
viscosity *μ*, Pa·s	0.1172*t*^−0.865^
density *ρ*, kg/m^3^	875.03 – 0.783*t* + 0.0012*t*^2^
specific heat *c*, J/(kg·K)	1767.0 + 4.122*t* + 0.0016*t*^2^
thermal conductivity *k*, W/(mK)	0.1232 – 2.55 × 10^−4^*t* + 1.25 × 10^−6^*t*^2^

**Table 3:** Specification of sensors, devices, and measuring apparatuses.

Measurement	Sensor	Measurement Range	Relative Uncertainty
Air flow rate [m^3^/s]	Kobold:		
	DMS111C4FD2	0–3.33 × 10^−4^	2.4%
	DMS214C4FD2	3.33 × 10^−4^–3.33 × 10^−3^	1.7%
Oil flow rate [m^3^/s]	Kobold:		
	KZA 1804R08	3.33 × 10^−7^–6.67 × 10^−5^	6%
Differential pressure [kPa]	Aplisens PR-28	10–50	0.7%
	Aplisens PR-28	50–150	0.4%
	Aplisens PC-28	0–600	0.3%
Temperature [°C]	K-type thermocouples	0–100	0.9%

**Table 4:** Selected operating conditions of the hydraulic motor.

Variant W-I					Variant W-II				
Designation	*p_AB_*, Pa	*t_g,in_*, °C	*n*, rev/min	*m_g_*·10^−3^, kg/s	Designation	*p_AB_*, Pa	*t_g,in_*, °C	*n*, rev/min	*m_g_*·10^−3^, kg/s
W-I.200	25 × 10^5^	−123	200	1.84	W-II.200	70 × 10^5^	−71	200	5.23
W-I.400			400	3.68	W-II.400			400	10.45
W-I.600			600	5.51	W-II.600			600	15.68
W-I.800			800	7.35	W-II.800			800	20.90
W-I.1000			1000	9.19	W-II.1000			1000	26.13
W-I.1200			1200	11.03	W-II.1200			1200	31.35
W-I.1400			1400	12.87	W-II.1400			1400	36.58
W-I.1600			1600	14.70	W-II.1600			1600	41.81
W-I.1800			1800	16.54	W-II.1800			1800	47.03

## Data Availability

The raw data supporting the conclusions of this article will be made available by the authors on request.

## Nomenclature

*a* *_sf_*	metal foam specific surface area, m^2^/m^3^
*A*	total heat transfer area, m^2^
*A_ch_*	channel area, m^2^
*A_sf_*	foam area, m^2^
*c*	specific heat, J/(kgK)
*d*	diameter of heat exchanger channel
*d_l_*	diameter of foam skeleton ligament, m
*d_p_*	pore diameter, m
*g*	mass flux, kg/(m^2^s)
*h*	heat transfer coefficient, W/(m^2^K)
*h_sf_*	interfacial convection heat transfer coefficient, W/(m^2^K)
*k*	thermal conductivity coefficient, W/(mK)
*k* *_eff_*	effective thermal conductivity coefficient, W/(mK)
*K*	foam permeability, m^2^
*m*	mass flow rate, kg/s
*n*	rotation speed, rev/min.
*Nu*	Nusselt number, (according to the Equations (8) and (9))
*p*	pressure, Pa
*p_AB_*	air pressure in converters of pneumatic–hydraulic motor
*Pr*	Prandtl number
*Re*	Reynolds number, (according to the Equation (10))
*q*	heat flux, W/m^2^
*Q*	heat/heat exchanger performance, W
*t*	temperature, °C
*T*	absolute temperature, K
*u*	velocity, m/s
*v_sg_*	air superficial velocity (related to the cross-section of the channel without filling), m/s
*V*	volume flow rate, m^3^/s
***Greek symbols***	
*β*	inertial coefficient, m
*δV_g_*	reduction in gas consumption, %
∆*p*/∆*l*	pressure drop, Pa/m
∆*t*	temperature difference, K
∆*t_g_*	air temperature increase, K
*ε*	porosity of metal foam,
*η*	efficiency of heat exchanger,
*μ*	viscosity, Pa∙s
*ρ*	density, kg/m^3^
*τ*	time, s
*ω*	pore density, PPI
***Subscripts***	
*b*	block of heat exchanger
*g*	gas/air
*cal*	calculated
*exp*	experimental
*in*	inlet condition
*ol*	oil
*out*	outlet condition

## References

[1] Shaw D., Yu J., Chieh C. (2013). Design of a hydraulic motor system driven by compressed air. Energies.

[2] Brol S., Czok R., Mróz P. (2020). Control of energy conversion and flow in hydraulic-pneumatic system. Energy.

[3] Mamala J., Brol S., Graba M. (2013). Hardware-in-the-loop type simulator of spark ignition engine control unit. Int. Symp. Electrodyn. Mechatron. Syst. SELM.

[4] Jantos J., Brol S., Mamala J. Problems in assessing road vehicle driveability parameters determined with the aid of accelerometer. Proceedings of the SAE World Congress & Exhibition.

[5] Mdi. https://air-volution.com.au/compressed-air-engine/.

[6] Regusci Air. http://regusciair.com/index.html.

[7] Prentice J. Tramway Information. http://www.tramwayinfo.com/Defair.htm.

[8] PSA Peugeot Citroën and Bosch Developing Hydraulic Hybrid Powertrain for Passenger Cars; 30% Reduction in Fuel Consumption in NEDC, up to 45% Urban; B-Segment Application in 2016, Website. https://www.greencarcongress.com/2013/01/psabosch-20130122.html.

[9] http://www.fleetowner.com/equipment/ups_hydraulic_hybrid_vehicles_1028.

[10] Brol S., Mamala J. Application of spectral and wavelet analysis in power train system diagnostic. Proceedings of the SAE World Congress & Exhibition.

[11] García-Moreno F. (2016). Commercial applications of metal foams: Their properties and production. Materials.

[12] Hassan A.M., Alwan A.A., Hamzah H.K. (2022). Metallic foam with cross flow heat exchanger: A review of parameters, performance, and challenges. Heat Transf..

[13] Hu H., Zhao Y., Li Y. (2023). Research progress on flow and heat transfer characteristics of fluids in metal foams. Renew. Sustain. Energy Rev..

[14] Kuruneru S.T.W., Vafai K., Sauret E., Gu Y.T. (2020). Application of porous metal foam heat exchangers and the implications of particulate fouling for energy-intensive industries. Chem. Eng. Sci..

[15] Hossain M.S., Shabani B. (2018). Experimental study on confined metal foam flow passage as compact heat exchanger surface. Int. Commun. Heat Mass Transf..

[16] Trilok G., Gnanasekaran N. (2021). Numerical study on maximizing heat transfer and minimizing flow resistance behavior of metal foams owing to their structural properties. Int. J. Therm. Sci..

[17] Jadhav P.H., Gnanasekaran N., Mobedi M. (2023). Analysis of functionally graded metal foams for the accomplishment of heat transfer enhancement under partially filled condition in a heat exchanger. Energy.

[18] Dongellini M., Naldi C., Cancellara S., Morini G.L. (2022). Experimental measurements of thermal–hydraulic performance of aluminum-foam water-to-air heat exchangers for a HVAC application. Appl. Therm. Eng..

[19] Fiedler T., Moore R., Movahedi N. (2021). Manufacturing and characterization of tube-filled ZA27 metal foam heat exchangers. Metals.

[20] Kim D.Y., Kim K.C. (2019). An experimental study on the thermal and hydraulic characteristics of open-cell nickel and copper foams for compact heat exchangers. Int. J. Heat Mass Transf..

[21] Samudre P., Kailas S.V. (2022). Thermal performance enhancement in open-pore metal foam and foam-fin heat sinks for electronics cooling. Appl. Therm. Eng..

[22] Arbak A., Dukhan N. (2020). Performance and heat transfer measurements in asymmetrically-heated metal foam cooled by water. Therm. Sci. Eng. Prog..

[23] Cicala G., Cirillo L., Diana A., Manca O., Nardini S. (2016). Experimental Evaluation of Fluid Dynamic and Thermal Behaviors in Compact Heat Exchanger with Aluminum Foam. Energy Procedia.

[24] Patil R., Shitole V.S., Lele M.M. (2020). Review on Metal Foam as Supreme Material for Air Cooling Heat Exchangers. Int. J. Res. Eng. Appl. Manag..

[25] Hamadouche A., Azzi A., Abboudi S., Nebbali R. (2018). Enhancement of heat exchanger thermal hydraulic performance using aluminum foam. Exp. Therm. Fluid Sci..

[26] Dyga R., Troniewski L. (2015). Convective heat transfer for fluids passing through aluminum foams. Arch. Thermodyn..

[27] Dyga R., Płaczek M., Witczak S. (2018). Influence of flow conditions and foam parameters on pressure drop and heat transfer in flow of fluids through channels filled with metal foams. MATEC Web Conf..

[28] Dyga R., Płaczek M. (2013). The permeability and inertia coefficient of open-cell aluminum foam. Chem. Eng. Equip..

[29] Brol S., Szegda A. (2018). Magnetism of automotive wheels with pneumatic radial tires. Meas. J. Int. Meas. Confed..

[30] JCGM Evaluation of Measurement Data—Guide to the Expression of Uncertainty in Measurement. 2008; 120p. https://www.bipm.org/utils/common/documents/jcgm/JCGM_100_2008_E.pdf.

[31] Tamkhade P.K., Lande R.D., Gurav R.B., Lele M.M. (2023). Investigations on tube in tube metal foam heat exchanger. Mater. Today Proc..

[32] Kumar K.K., Kotresha B., Naik K. (2023). Flow and heat transfer irreversibility in partial filled metal foams. Int. J. Therm. Sci..

[33] Xu Z.G., Gong Q. (2018). Numerical investigation on forced convection of tubes partially filled with composite metal foams under local thermal non-equilibrium condition. Int. J. Therm. Sci..

[34] Bhattacharya A., Calmidi V.V., Mahajan R.L. (2002). Thermophysical properties of high porosity metal foams. Int. J. Heat Mass Transf..

[35] Buonomo B., Di Pasqua A., Manca O., Nappo S. (2021). Numerical Study on Thermal and Fluid Dynamic Behavior of Confined Impinging Slot Jets with Nanofluids in Partially Filled Configuration of Metal Foam. J. Phys. Conf. Ser..

[36] Chen K., Wang X., Chen P., Wen L. (2022). Numerical simulation study on heat transfer enhancement of a heat exchanger wrapped with metal foam. Energy Rep..

[37] Ansys Fluent User’s Guide v. (2021). 2021/R2. https://ansyshelp.ansys.com/account/secured?returnurl=/Views/Secured/corp/v242/en/flu_th/flu_th.html.

[38] Huisseune H., De Schampheleire S., Ameel B., De Paepe M. (2015). Comparison of metal foam heat exchangers to a finned heat exchanger for low Reynolds number applications. Int. J. Heat Mass Transf..

[39] Buonomo B., di Pasqua A., Manca O., Nardini S. (2020). Evaluation of thermal and fluid dynamic performance parameters in aluminum foam compact heat exchangers. Appl. Therm. Eng..

[40] Li Y., Gong L., Ding B., Xu M., Joshi Y. (2021). Thermal management of power electronics with liquid cooled metal foam heat sink. Int. J. Therm. Sci..

[41] Xu Z.G., Qin J., Zhou X., Xu H.J. (2018). Forced convective heat transfer of tubes sintered with partially-filled gradient metal foams (GMFs) considering local thermal non-equilibrium effect. Appl. Therm. Eng..

[42] Xu H., Gong L., Huang S., Xu M. (2015). Flow and heat transfer characteristics of nanofluid flowing through metal foams. Int. J. Heat Mass Transf..

[43] Zhukauskas A., Hartnett J.P., Irvine T.F. (1972). Heat Transfer from Tubes in Crossflow. Advances in Heat Transfer.

